# Finite element modeling and experimental validation of wedge fractures in the thoracic spine

**DOI:** 10.1007/s43390-026-01276-3

**Published:** 2026-01-18

**Authors:** Sacha Guitteny, Rana A. Ahmad, Abdullah Memon, Julie Mikhail, Michael J. Patetta, Steven M. Mardjetko, Farid Amirouche

**Affiliations:** 1 Department of Orthopaedic Surgery, University of Illinois College of Medicine at Chicago, Chicago, IL 60607 USA; 2 Orthopaedic and Spine Institute, Northshore University HealthSystem, Northshore University HealthSystem, 9669 Kenton Avenue, Suite 305, Skokie, IL 60076 USA

**Keywords:** Finite elements, Vertebral compression fractures, Wedge fractures, Kyphoplasty, Vertebroplasty, Spinal deformity

## Abstract

**Purpose:**

Vertebral compression fractures (VCFs) are the most common fractures in patients with osteoporosis, contributing to approximately 700,000 spinal fractures annually. Wedge fractures, characterized by anterior vertebral body collapse, are the most prevalent type of VCFs and a significant cause of spinal deformity, such as thoracic kyphosis. This study aimed to develop and validate a finite element model (FEM) of wedge fractures to understand their biomechanics and clinical implications, and help future studies to elucidate spine fractures in osteoporotic patients.

**Methods:**

CT-based finite element models of T9–T12 vertebral bodies were developed using scans from four cadaveric spines. Axial compression tests were performed on the corresponding vertebrae using a Materials Test Systems (MTS) machine to induce wedge fractures. Key biomechanical parameters, including stiffness and strength, were measured and compared to FEM predictions for validation.

**Results:**

The FEM demonstrated strong agreement with the experimental data, achieving coefficients of determination (*R*^2^) of 0.71 (*p* < 0.01) for stiffness and 0.88 (*p* < 0.01) for strength. The FEM predicted a stiffness of 5.9 ± 0.6 kN/mm and a strength of 3.2 ± 0.4 kN, which closely matched the experimental values of 5.83 ± 1.2 kN/mm and 3.54 ± 0.6 kN, respectively. The FEM also qualitatively reproduced fracture patterns, including mid-fracture lines and delamination of the anterior cortical shell.

**Conclusions:**

This study validates FEM as a robust tool for modeling wedge fractures and understanding their role in spinal deformity. The model offers insight into vertebral compression fractures and can be further developed for use in other clinical applications, to provide the volume needed to restore the height of the vertebrae**.**

## Introduction

**Fig. 1:**
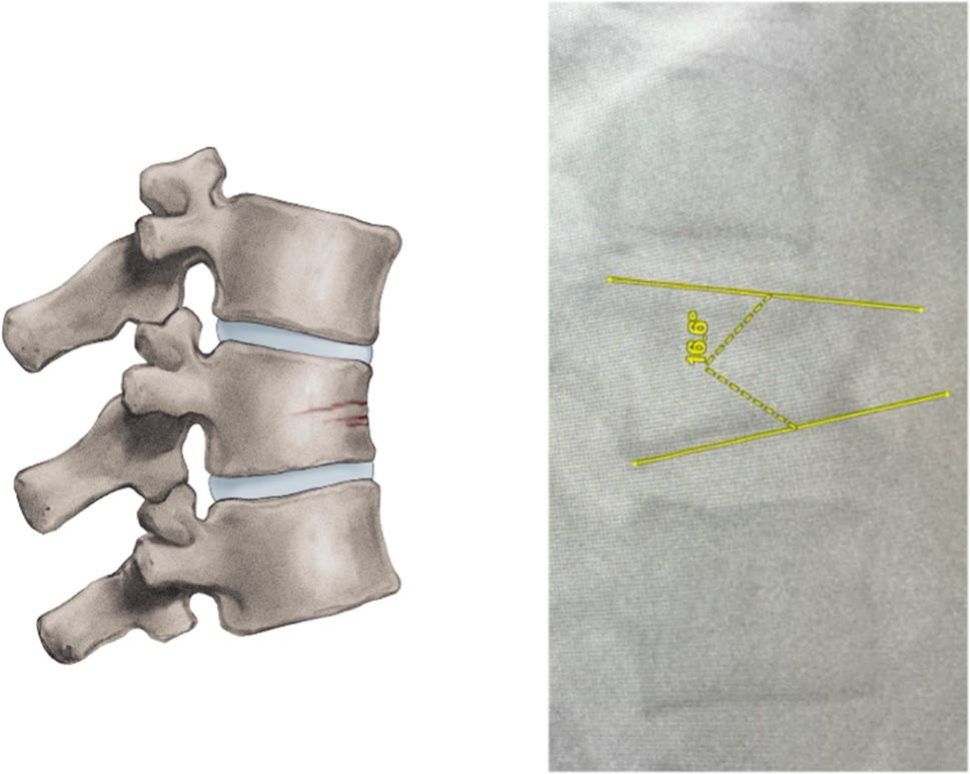
Wedge compression fracture animation

Vertebral compression fractures (VCFs) are the most common type of fracture for patients with osteoporosis, comprising about 700,000 out of 1.5 million spine fractures [1]. These injuries can range in severity from mild, causing minimal pain and deformity, to severe, resulting in intense pain, sagittal imbalance, and potential neurological deficits [1, 2]. VCFs can be categorized by fracture pattern: biconcave, burst, crush, or wedge. Wedge fractures are the most common type of VCFs and are commonly associated with osteoporosis and minor trauma [3, 4]. The pattern of wedge fracture results in the anterior portion of the vertebral body collapsing and the posterior component remaining intact, resulting in a “wedge” shape (Fig. 1). Wedge fractures often can progress to spine deformity, particularly kyphotic deformity [5].

The thoracolumbar junction is the most common site for wedge fractures due to its unique anatomical and biomechanical properties. As a transition zone between the rigid thoracic spine and the more flexible lumbar spine, this region is subjected to concentrated mechanical stresses, making it particularly vulnerable to anterior vertebral collapse. Furthermore, the anterior vertebral bodies in this region are structurally weaker, predisposing them to wedge-shaped deformities under compressive forces [6]. This prevalence is especially pronounced in osteoporotic patients, where diminished bone density further increases the risk of fracture from low-energy trauma.

VCFs, particularly in older adults, are associated with significant morbidity, including chronic pain, functional impairment, kyphotic deformity, and pulmonary complications. In severe cases, these fractures can contribute to increased mortality due to secondary health risks such as immobility-related complications, respiratory compromise, and the development of a fracture cascade [7]. Early diagnosis and effective management are critical to mitigating these outcomes.

Wedge fractures are further complicated by their impact on adjacent vertebrae, particularly when anterior wedging exceeds 50%. This condition becomes even more critical in the presence of severe hyper-kyphosis, which can exacerbate spinal deformity and functional impairment. Wedge fractures result in a loss of vertebral body height, with the degree of height loss playing a key role in determining the appropriate course of treatment. For height loss of 10–30%, conservative management, including bracing and pain control, is typically sufficient. Height loss between 30 and 50% may warrant escalated treatment, potentially involving minimally invasive interventions. When vertebral height loss exceeds 50%, surgical intervention is often necessary to ensure adequate spinal stability and acceptable sagittal alignment. [8–13].

Finite element modeling (FEM) offers a powerful tool for enhancing vertebroplasty and kyphoplasty by simulating the biomechanical environment of the spine and optimizing procedural parameters. FEM can incorporate patient-specific factors such as bone density and fracture geometry to model stress distribution and predict outcomes. For vertebroplasty, FEM can guide selection of cement type, volume, and injection pressure to achieve optimal stabilization while minimizing the risk of leakage and adjacent-segment fractures [14, 15]. In kyphoplasty, FEM can simulate balloon inflation effects, enabling precise correction of height and redistribution of spinal stresses [16, 17]. Additionally, FEM can predict long-term outcomes, such as fatigue resistance and stability under repetitive loading, allowing for more personalized and effective treatment planning. By reducing complications and improving procedural accuracy, FEM has the potential to transform the management of vertebral compression fractures.

Previous studies have shown that the thoracolumbar region is more susceptible to VCFs due to the transition from a rigid thoracic spine to a more flexible lumbar spine. This study specifically focused on the lower thoracic vertebrae (T9–T12). This segment represents a critical transitional zone between the rigid thoracic spine and the more mobile lumbar spine, making it particularly susceptible to biomechanical stresses and fractures. Analyzing this region offers clinically relevant insights into the biomechanics of wedge fractures, as these injuries frequently result in significant sagittal imbalance and kyphotic deformity. Furthermore, fractures in the T9–T12 region are representative of injury mechanisms at the thoracolumbar junction and provide a practical, focused approach for validating finite element models. This focus avoids excessive variability that could arise from including lumbar vertebral bodies with distinct biomechanical properties.

The objective of this study was to create a CT-based FEM model of the vertebral bodies T9–T12 from four cadavers to simulate wedge fractures. This FEM model was validated through physical experimental fractures by subjecting the cadaveric vertebral bodies to an axial force using the follower load technique [18] until the bone fractured. Each vertebral body was separated from the others, and an external force was applied, similar to a fall or a sudden heavy-weight load. Stiffness and peak load measurements from the experimental fractures were then compared with FEM predictions to ensure model accuracy.

## Materials and methods

### Specimen dissection and preparation

This study used vertebral bodies T9–T12 from four cadaveric spines (16 vertebral bodies). The characteristics of these samples are summarized in Table 1. Each vertebra was separated from the others. All soft tissues, including muscles, ligaments, and intervertebral discs, were removed. The cartilaginous endplates were partially removed to obtain the plane surfaces required for the axial compression test, and the surfaces were polished to ensure proper loading conditions. The final vertebra’s thickness allowed for a layer of cortical bone on the upper and lower surfaces. The posterior elements of the vertebrae were also removed. This preparation process was conducted to ensure that an experimental axial load would result in a wedge fracture.

**Table 1:** Demographics of the subjects from which the vertebrae were extracted

Spine	Age	Sex	Vertebrae level
Spine 1	93	F	T9 to T12
Spine 2	67	M	T9 to T12
Spine 3	61	F	T9 to T12
Spine 4	73	F	T9 to T12

### CT modeling

**Fig. 2:**
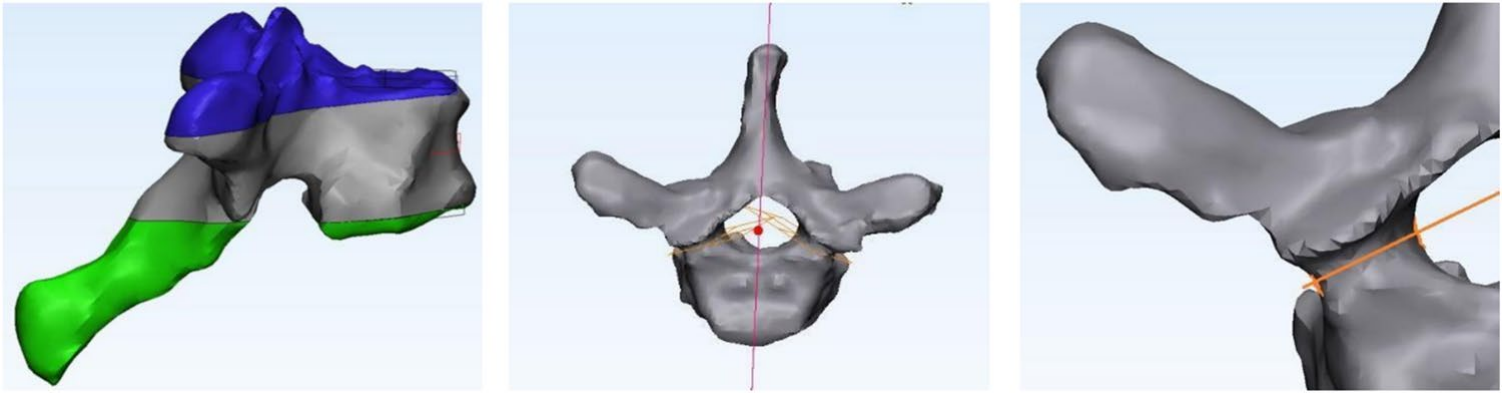
Vertebral planes are defined for each 3D reconstruction from MIMICS and 3-MATIC

All 16 vertebral bodies were scanned using computed tomography (CT) imaging to develop the finite element model (FEM). The CT imaging specifications are provided in Table 2. MIMICS software was used to create 3D models from the CT scans of the vertebral bodies. These 3D models were then refined in 3-MATIC software to generate high-quality surface meshes and define reference planes (Fig. 2) for the FEM simulation. A transverse plane was defined as the midpoint between the cranial and caudal surfaces of each vertebral body, corresponding to the superior and inferior endplates. The sagittal plane was aligned with the spinous process and passed through the vertebral center of mass, following the curvature of the pedicle. The resulting mesh consisted of tetrahedral finite elements with an edge length of approximately 1 mm, providing a detailed, anatomically accurate representation for simulation.

**Table 2:** Specifications defined for CT scan

X-ray tube current	200 mA
KVP	120 kV
Slice thickness	max = 0.5 mm
Pixel slice	max = 0.27 mm
Slice width	min = 512 px
Slice height	min = 512 px

**Fig. 3:**
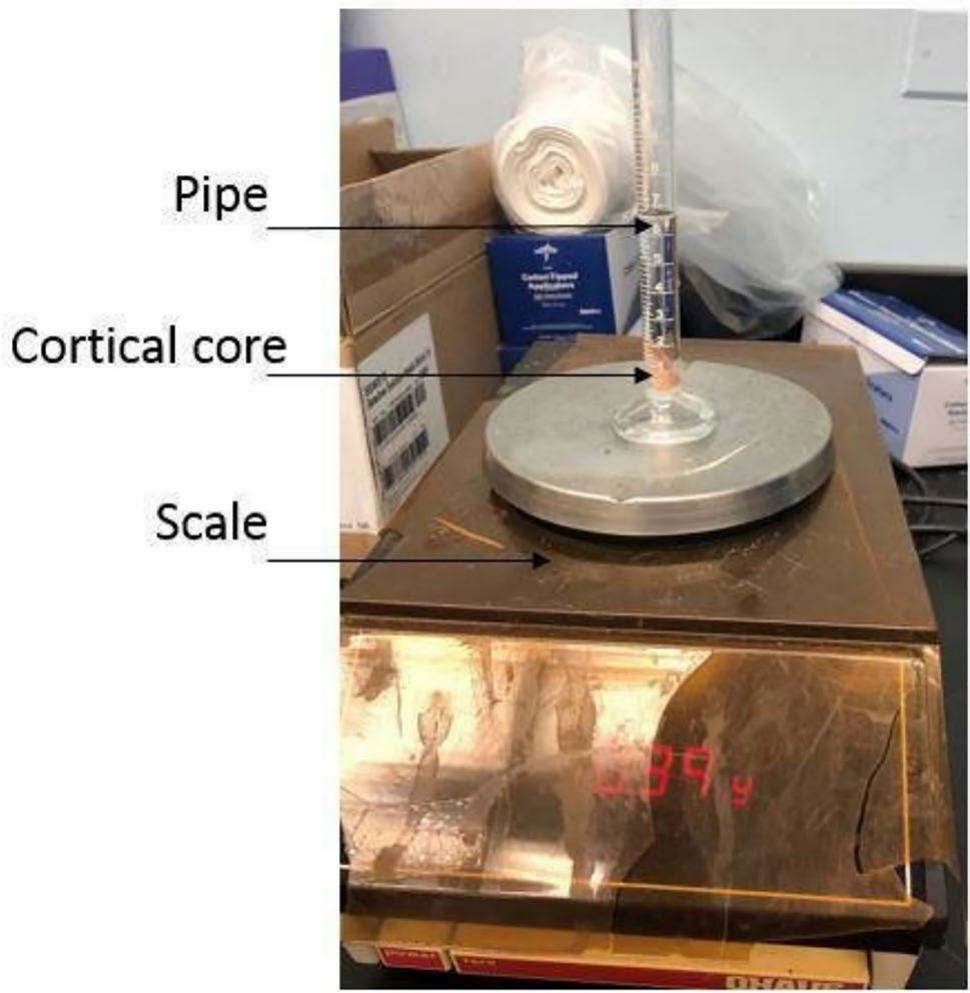
Density test—weight and volume scaling of a vertebral cortical core

Next, CT gray-scale values were converted to physical density values. This conversion was done using a method previously described by Silva et al. [19]. Density tests were conducted on 16 cortical cores from the lower and upper facets of the vertebra using Eq. (1) (Fig. 3). The constants *a* and *b* were determined by restricting the CT minimum gray-scale value to 0.01 g/cm^3^ density to prevent negative density values (Table 3). Here, *ρ* signifies density, and HU is the abbreviation for Hounsfield Units

1 $$\rho = a + HU*b.$$

**Table 3:** Constants assigned to the material properties of cadaver models for the range of FE

*a*	*b*	Number of finite elements (FE)
0.32	0.0003	161,213–336,620

The material assignments to the finite elements were assumed to be isotropic. Using MIMICS, Eq. (2) was computed to define Young’s modulus (*E*) as a function of density *ρ*, with a ratio of *ρ*_app_/*ρ*_ash_ equal to 0.6. The yield strain (*ε*_*y*_) was defined as a function of density *ρ* (Eq. (3)). Poisson’s ratio for each element was set at $\nu$ = 0.3 [20]

2 $$E = 3050\rho^{1.81}$$

3 $$\varepsilon_{y} = 0.0065\rho^{ - 1.42} .$$

The 3D models were edited in ABAQUS software to incorporate plastic behavior. For each finite element, the post-yield behavior was set as plastic and is described by Eq. (4) with σ (constraint) defined as follows:

4 $$\sigma = E\varepsilon if \sigma < \sigma_{y} , \sigma_{y} if \sigma \ge \sigma_{y} .$$

**Fig. 4:**
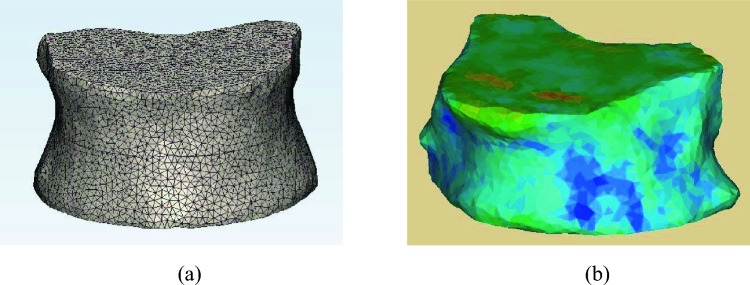
**a** Vertebral body finite element model with refined mesh and **b** its material assignment map

An example of a vertebral body’s mesh and corresponding material assignment is shown in Fig. 4.

### Finite element analysis (FEA)

**Fig. 5:**
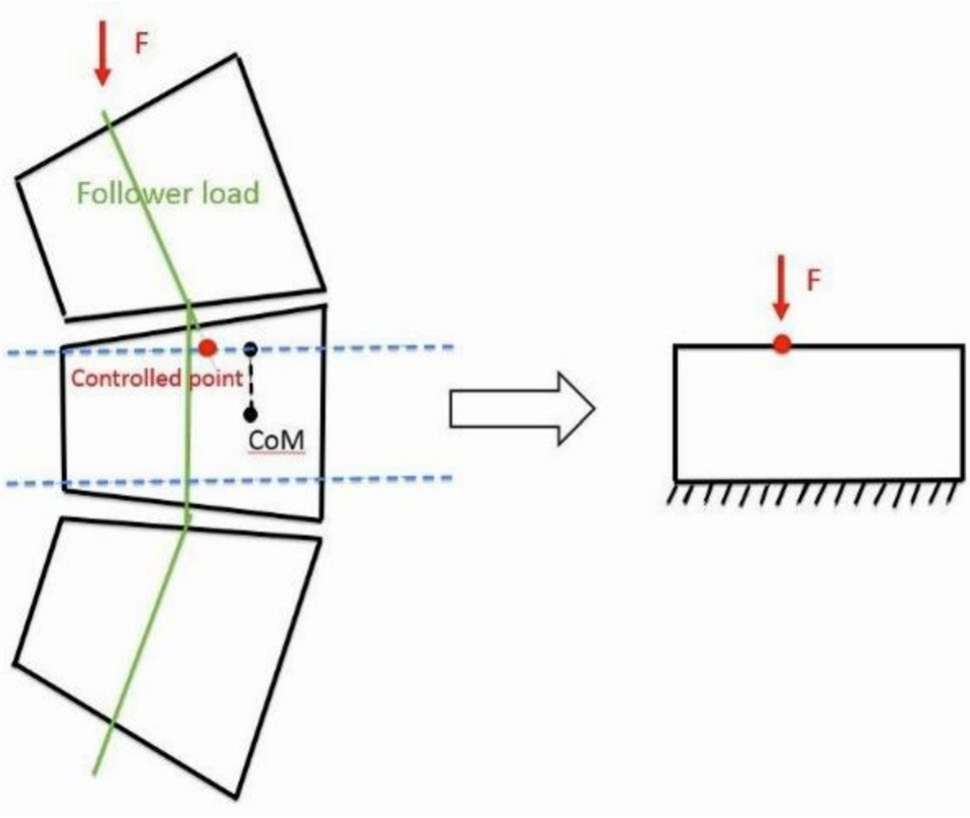
Mechanical test performed on cadaver vertebrae: follower load force compared to pure axial compression

Finite element analysis (FEA) was conducted using ABAQUS software. The vertebral model was constrained to pure axial compression, with both static and nonlinear analyses. The follower load technique [18] was employed to determine the controlled point displacement from the center of mass, which served as the optimal location for applying compression to simulate an anterior wedge fracture of the vertebral body (Fig. 5). Multiple Point Constraints (MPCs) were used to link the controlled point with the nodes on the cranial surface of the vertebra. These MPCs ensured that the cranial surface functioned as a reference plane with zero displacement.

FEM analyses were performed to generate a load–displacement curve. Stiffness was calculated as the slope of the linear region of the load–displacement curve, while strength was defined as the maximum load within the elastic response domain. For qualitative analysis, the failure pattern was identified by locating fracture lines, which were visualized by highlighting finite elements exhibiting nonzero plastic strain.

### Experimental testing

**Fig. 6:**
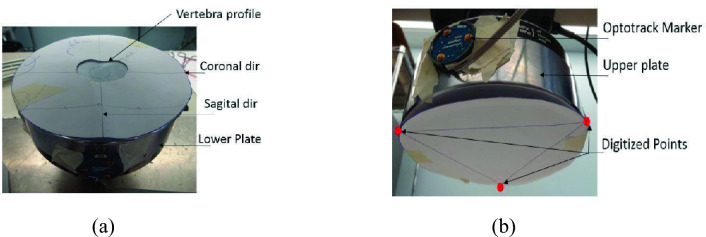
**a** The procedure for positioning the samples in the testing machine is represented. **b** The axial displacement was computed via three digitized points in the compression testing setup

The experimental compression of the vertebral bodies was performed using a Material Testing Systems (MTS) tensile machine equipped with compression steel plates. The upper loading plate was designed to allow rotation of up to 5° relative to the MTS connector. Proper specimen placement in the MTS machine was achieved by ensuring that the center of the loading plate corresponded to the desired loading point on the vertebral body (10% of the body’s width from the center of mass in the sagittal direction). This alignment was facilitated by marking the lower profile of the vertebral body on the lower compression plate. The vertebral body’s lower profile was reconstructed using 3D-printed circular plastic pieces. Points were defined in the coronal and sagittal planes, and a hole corresponding to the vertebral body profile was created in the plastic piece using laser cutting. This process ensured accurate alignment of the vertebral bodies within the MTS machine, enabling precise delivery of the axial compression force at the desired point. A pure axial load was applied at a quasi-static displacement rate of 3 mm/min and continued until complete failure of the vertebra (Fig. 6).

During compression testing, load values were recorded using the MTS cells, while vertebral body motion on the upper loading plate was tracked using OPTOTRAK technology. The motion of the upper loading plate was monitored by tracking its coordinates, enabling precise measurement of the displacement at the center point. These data were combined to generate the load–displacement curve, which provides a comprehensive representation of the vertebral body's mechanical response under axial compression.

## Results

### Experimental testing results

**Fig. 7:**
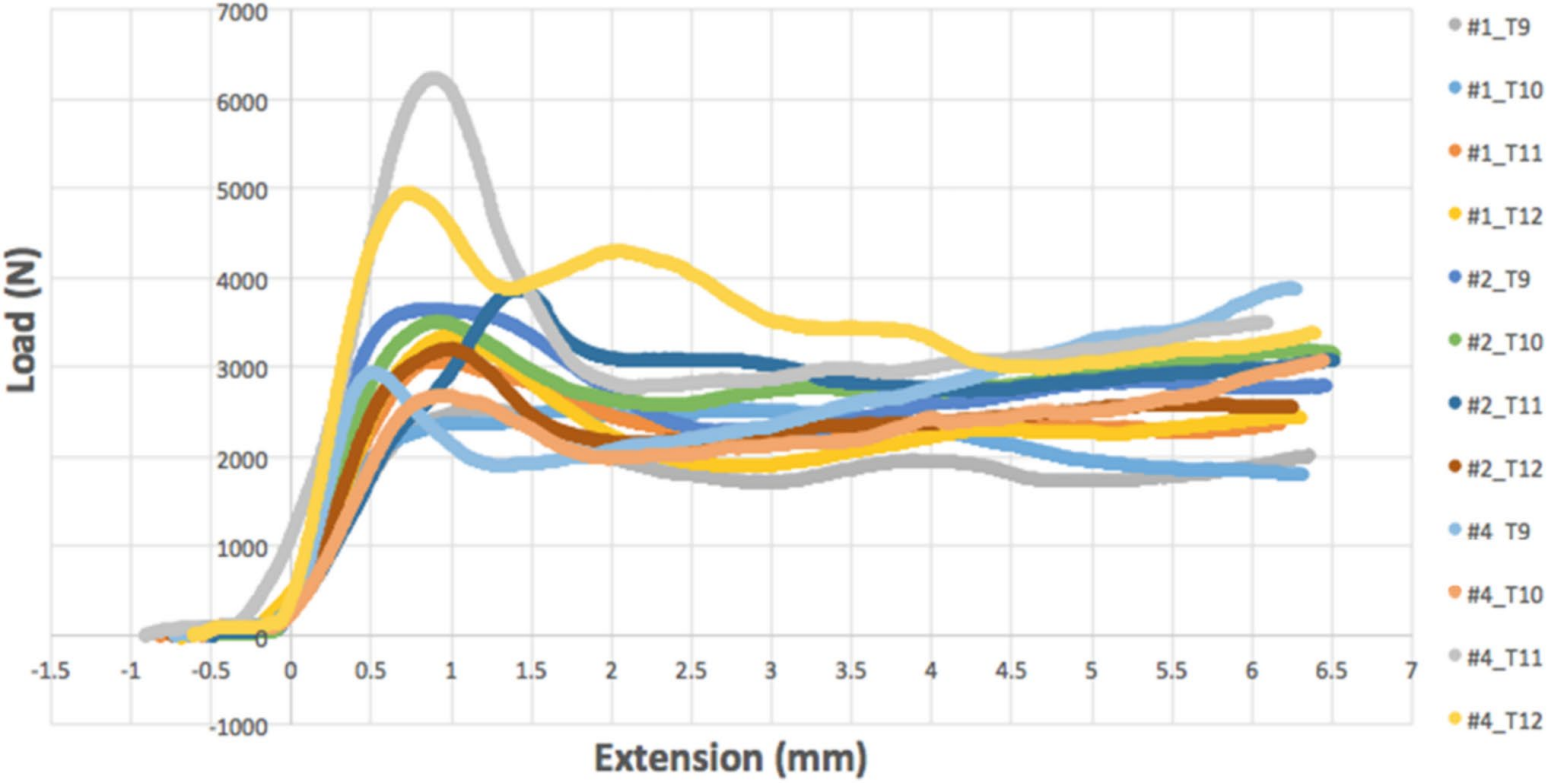
Load–displacement curves from the vertebrae compression tests

The four cadaveric spines were assigned numerical identifiers (1–4). Spine 3 was excluded from the analysis due to severe osteoporosis, as indicated by pre-test radiological and gross morphological features (marked cortical thinning and reduced trabecular density). This condition contributed to significant deviations in mechanical properties (stiffness error = 79.6%; peak load error = 58.4%). The load–displacement curves generated from the experimental compressions are shown in Fig. 7. The average stiffness of the vertebral bodies was 5.83 ± 1.2 kN/mm (range: 3.6–9.5 kN/mm), and the average strength was 3.54 ± 0.6 kN (range: 2.53–6.23 kN). The average error for stiffness and strength values was 34% and 23%, respectively.

**Fig. 8:**
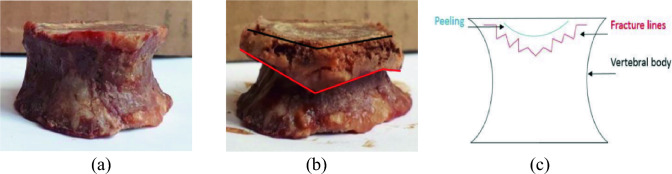
Intact **a** and fractured **b** T10 vertebral body from spine 4 in the anterior view with peeling (black line) and fracture pattern (red line); explicative scheme **c**

Qualitative fracture patterns are depicted in Fig. 8, showing the intact (8a) and fractured (8b) T10 vertebral body of spine 4. The fractured vertebrae exhibit upper delamination, with the cortical shell peeling along the edges. A red fracture line indicates the failure pattern observed under pure axial compression, showing separation of the anterior cortical shell from the superior and inferior surfaces. The maximum tilt during compression was 1.73° for the vertebral bodies.

### FEM analysis

The strength and stiffness values generated from the FEM analysis were compared to the experimental compression results using linear correlation. While the yield strain ($\varepsilon_{y}$) from the FEM model was consistent with the experimental data, the average stiffness and strength values were significantly higher in the FEM model, resulting in increased error. This discrepancy is likely attributable to the osteoporotic changes in the sample vertebral bodies used for the experimental compression tests.

**Fig. 9:**
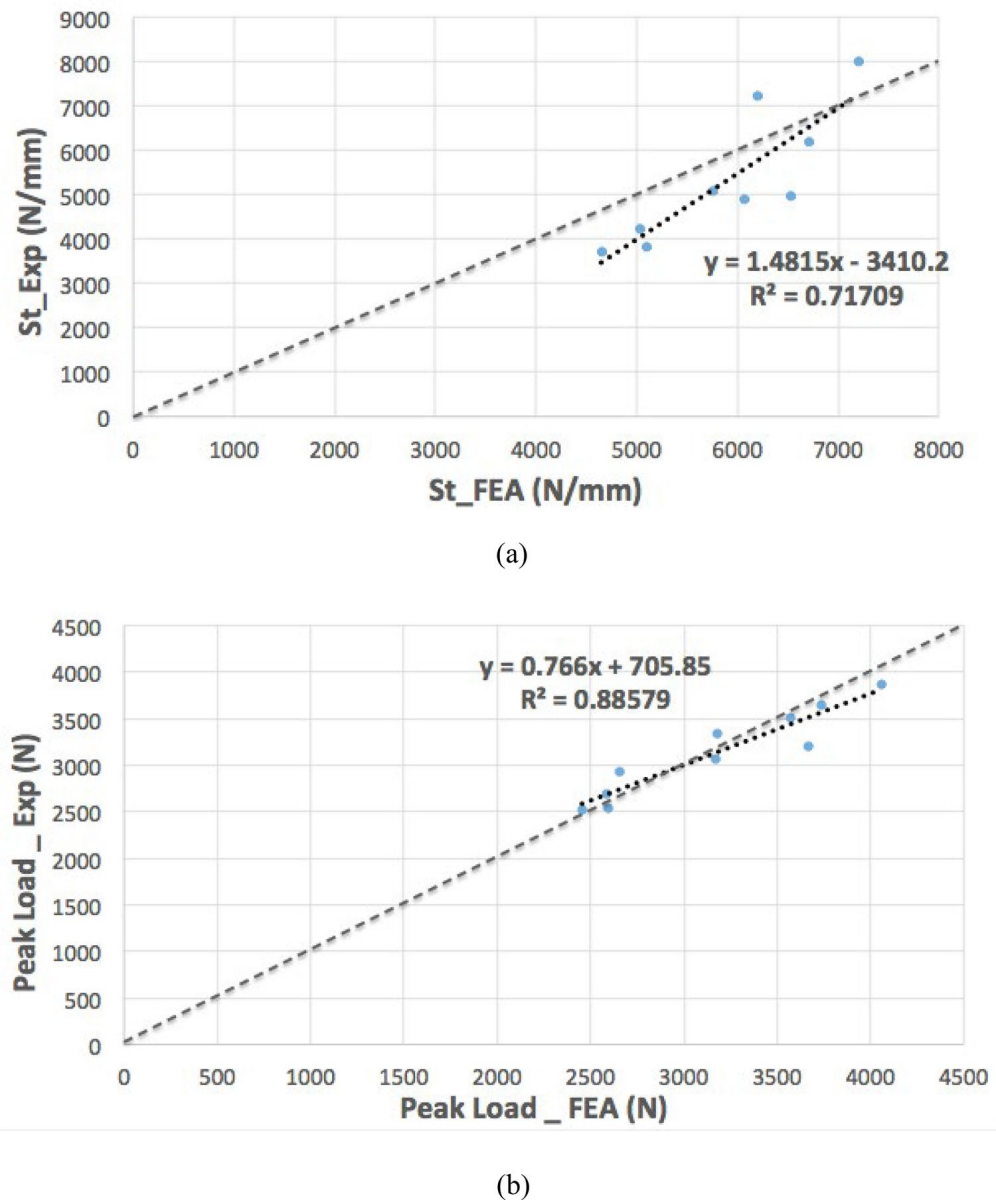
Stiffness **a** and Peak Load **b** predictions with the FEM model. The dashed line represents the quadrant bisector

To address this issue, the Young’s modulus of the FEM model was scaled down by a factor of 1.85. This adjustment resulted in average stiffness and strength values of 5.9 ± 0.6 kN/mm (range: 4.6–7.2 kN/mm) and 3.2 ± 0.4 kN (range: 2.5–4.1 kN), respectively, aligning more closely with the experimental results. This scaling significantly reduced errors in stiffness from 83.3% to 19.6% (range: 7.9%–32.7%) and in strength to 5.0% (range: 1.6%–14.5%). These adjustments yielded high coefficients of determination (*R*^2^) for both stiffness (*R*^2^ = 0.71, *p* < 0.01) and strength (*R*^2^ = 0.88, *p* < 0.01), indicating a strong, statistically significant correlation between the FEM model predictions and the experimental compression results (Fig. 9).

**Fig. 10:**
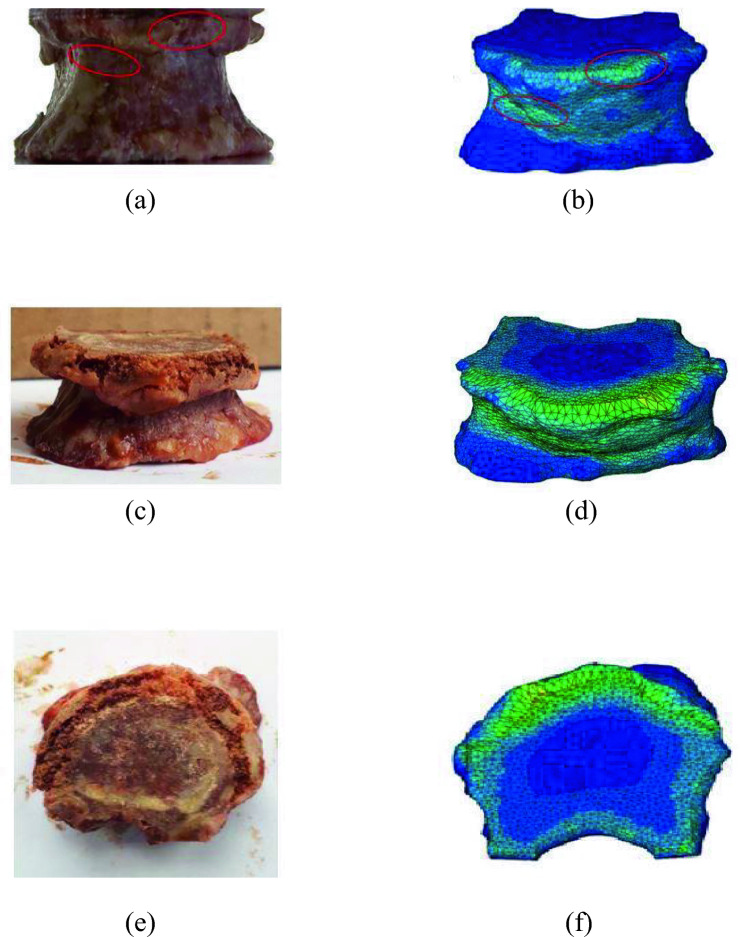
Qualitative comparison between the experimental damage after 4.5 mm compression (a, b) and compression to failure (c, d, e, f) for T10 specimen

Furthermore, the failure pattern observed in the experimental images was qualitatively compared to the regions of nonzero equivalent plastic strain predicted by the FEM. The finite element model provided a good qualitative description of the damage pattern. Specifically, the creation of the mid-fracture line, its distribution along the anterior wall, and the resulting damage localized at the top of the anterior cortical shell were consistent between the experiments and the FEM. These observations are illustrated in Fig. 10.

## Discussion

Wedge compression fractures are among the most common vertebral pathologies, often leading to significant clinical challenges. Given the prevalence of wedge fractures and their contribution to spinal deformities, this validated FEM model offers a promising tool for guiding procedural planning and optimizing parameters for spine-stabilizing interventions. By simulating fracture behavior under physiological loading conditions, this study provides critical insights to improve treatment outcomes and reduce complications associated with spine deformities.

The objective of this study was to validate a CT-based FEM model of wedge fractures by evaluating the stiffness and strength associated with vertebral failure. To achieve this, 16 cadaveric vertebral bodies underwent axial compression testing, and the results were used to validate the FEM model for T9–T12. The FEM accurately predicted stiffness (5.9 ± 0.6 kN/mm) and strength (3.2 ± 0.4 kN) after scaling Young’s modulus, reducing errors to 19.6% for stiffness and 5% for strength. The calculated coefficient of determination of *R*^*2*^ = 0.71 (*p* < 0.01) for stiffness and *R*^*2*^ = 0.88 (*p* < 0.01) for strength. Furthermore, the model successfully reproduced experimentally observed fracture patterns, including the mid-fracture line and anterior cortical shell delamination. Thus, this FEM model demonstrates utility in simulating wedge fractures of the lower thoracic spine.

Over the past decade, finite element modeling has become an invaluable tool for investigating vertebral compression fractures (VCFs), particularly in the thoracolumbar spine [21–25]. However, relatively few studies have specifically addressed the lower thoracic vertebrae (T9–T12), which represent a common site of clinically significant wedge fractures. Fei et al. developed a 3D FEM model of the T10–L2 segment in an elderly female with a single T12 osteoporotic VCF, demonstrating increasing vertebral stress under axial loading [26]. Our study extends this work by validating FEM predictions of strength, stiffness, and wedge deformity for multiple lower thoracic vertebrae using cadaveric specimens. Similarly, Giambini et al. employed an extended FEM (X-FEM) model derived from quantitative CT of three cadaveric L3 vertebrae, reporting a strong correlation between FEM-predicted and experimentally measured failure load and stiffness [20]. Our findings demonstrate a comparable correlation for lower thoracic vertebrae, supporting the applicability of FEM to this region of the spine.

Several prior studies have validated FEM models of the thoracolumbar spine against experimental data. For example, Silva et al. reported an excellent correlation for yield loads (r^2^ > 0.86) but a more variable correlation for stiffness (r^2^ = 0.58–0.79) [19], a pattern consistent with our findings. Other studies reported similar findings for strength and stiffness when validating their CT-based FEM models of the thoracolumbar region [22, 24, 27]. Our study focuses specifically on the lower thoracic spine (T9–T12) and, through a strong correlation between FEM predictions and experimentally induced fractures, supports the use of FEM in this anatomically and clinically important region.

The validated FEM model provides a robust tool for understanding the biomechanics of wedge fractures and guiding spine-stabilizing procedures such as vertebroplasty and kyphoplasty [28, 29]. By simulating the fracture mechanics and stress distribution within the vertebra, the FEM model can guide the optimal placement and volume of cement for both procedures. Additionally, the model's ability to predict fracture patterns and stress redistribution provides valuable insights for minimizing complications, such as adjacent-segment fractures, which are more common after vertebroplasty [30]. For kyphoplasty, the FEM can be used to evaluate the effectiveness of height restoration and kyphosis correction under different loading conditions.

This integration of FEM into clinical decision-making can enhance the precision and safety of spinal deformity wedge fractures, ensuring that treatments are tailored to the specific biomechanical and anatomical characteristics of the fractured vertebra. Future studies could extend the application of this model to optimize procedural parameters and improve outcomes in patients with complex spinal deformities.

This study had some limitations. First, bone densities for the experimental data were estimated by dividing the vertebrae's mass by their volume, which may not provide the most accurate representation of bone quality. Dual X-ray absorptiometry (DEXA), a gold standard for assessing bone mineral density, could be used in future studies to achieve more precise measurements [31]. Second, the experimental setup allowed the upper plate to be tilted or rotated by less than 7° due to the loose plate attachment. Incorporating a ball joint, as suggested by Dall’Ara et al. [27], could improve the validity of the setup by more closely replicating real-world loading conditions and fracture mechanics. Third, the cross-sectional imaging used in this study could be enhanced by thinner-section acquisitions to capture native bone microstructure better, thereby improving the accuracy of finite element simulations [32, 33]. Finally, the age of the cadavers ranged from 61 to 93 years, with three of the four specimens being female. These characteristics increase the likelihood that the spines exhibited osteoporotic changes, which may have influenced the experimental results and contributed to the observed variability in stiffness and strength. While this represents a common clinical population for wedge fractures, it may limit the model's generalizability to younger, non-osteoporotic individuals. Future studies should include a more diverse sample across age, sex, and bone health to improve the model's robustness and applicability across different patient demographics.

## Conclusions

Wedge fractures, a prevalent form of vertebral compression fractures (VCFs), are a significant contributor to spinal deformities such as thoracic kyphosis. Understanding and predicting the biomechanics of these fractures are critical for improving clinical outcomes, particularly in patients at risk for progressive deformity. This study developed and validated a CT-based finite element model (FEM) of the lower thoracic vertebrae (T9–T12) that accurately predicts stiffness, strength, and fracture patterns. By incorporating experimental compression data, the model reproduced clinically relevant features, including mid-fracture lines and anterior cortical shell delamination.

The relevance of this model lies in its potential to guide the management of VCFs. It provides a powerful tool for predicting fracture mechanics, understanding deformity progression, and optimizing spine-stabilizing procedures, such as vertebroplasty and kyphoplasty. In clinical practice, this model could help personalize interventions and enable providers to predict the endplate's response to interventional corrective procedures.

Thus, this study represents an important step toward bridging the gap between computational modeling and clinical application. By simulating the biomechanical behavior of wedge fractures with high fidelity, FEM offers new opportunities to advance the prevention and treatment of spinal deformities. Future studies building on this model could expand its scope to include diverse patient populations, patient-specific modeling, and real-time clinical applications, further solidifying its utility in the management of spinal pathologies.

## Data Availability

Readers can contact the corresponding author for specific information if needed.
